# Boosting CO_2_ hydrogenation to methane over Ni-based ETS-10 zeolite catalyst

**DOI:** 10.3389/fchem.2022.1041843

**Published:** 2022-10-11

**Authors:** Mei Xiang, Zhangxi Gao, Xiaonan Ji, Dantong Li, Yaoyao Deng, Yalong Ding, Chi Yu, Wei Zhang, Zhenwei Zhang, Zeying Wu, Jiancheng Zhou

**Affiliations:** ^1^ Research Center of Secondary Resources and Environment, School of Chemical Engineering and Materials, Changzhou Institute of Technology, Changzhou, China; ^2^ Department of Chemical Engineering, School of Chemistry and Chemical Engineering, Southeast University, Nanjing, China; ^3^ Jiangsu Key Laboratory of Advanced Catalytic Materials and Technology, Changzhou University, Changzhou, China; ^4^ College of Chemistry and Pharmaceutical Engineering, Huanghuai University, Zhumadian, China; ^5^ Zhongyi Testing and Research Institute Co, Ltd., Huzhou, China

**Keywords:** CO_2_ hydrogenation, methane, ETS-10 zeolite, *in situ* doping, Ni-based catalyst

## Abstract

The activation and conversion of the CO_2_ molecule have always been the most vexing challenge due to its chemical inertness. Developing highly active catalysts, which could overcome dynamic limitations, has emerged as a provable and effective method to promote CO_2_ activation–conversion. Herein, ETS-10 zeolite–based catalysts, with active nickel species introduced by *in situ* doping and impregnation, have been employed for CO_2_ methanation. Conspicuous CO_2_ conversion (39.7%) and perfect CH_4_ selectivity (100%) were achieved over the Ni-doped ETS-10 zeolite catalyst at 280°C. Comprehensive analysis, which include X-ray diffraction, N_2_ adsorption–desorption, SEM, TEM, H_2_ chemisorption, CO_2_ temperature programmed desorption, and X-ray photoelectron spectroscopy, was performed. Also, the results indicated that the resultant hierarchical structure, high metal dispersion, and excellent CO_2_ adsorption–activation capacity of the Ni-doped ETS-10 zeolite catalyst played a dominant role in promoting CO_2_ conversion and product selectivity.

## 1 Introduction

As one of the major greenhouse gases, CO_2_ has commanded the attention of the whole world due to its increasing emission that results in a series of critical environmental problems (Saeidi et al., 2021). While being not blamed for the ecological concern, CO_2_ is in fact quite an important C1 source to produce high value–added chemicals, such as CH_4_, CH_3_OH, HCOOH, CH_3_CH_2_OH, and other C^2+^ products (Wang et al., 2021; Zhu et al., 2022). Hence, extensive and continuous efforts have been made to reduce CO_2_ emissions, among which catalytic hydrogenation has been proved to be an attractive and promising process in which the required hydrogen is produced renewably by water electrolysis (Meng et al., 2022). In particular, the hydrogenation of CO_2_ into methane has been identified as one of the most important and economically feasible strategies, during which the produced simplest C-H molecule has high gravimetric/volumetric energy density and is easily liquefied and safely transported by means of the existing natural gas infrastructure (Zhao et al., 2022). However, due to the chemical inertness of the CO_2_ molecule, its activation and conversion have always been the most vexing challenges. The current solutions cannot be divorced from the use of high energy consumption and an efficient catalyst. Therefore, a wide range of studies have been focused on developing highly active catalysts that are conducive in overcoming dynamic limitations and promoting CO_2_ activation–transformation (Gao et al., 2022; Liu et al., 2022; Song et al., 2022).

Bifunctional catalysts, consisting of active metal components and supports, are the most widely employed and investigated for CO_2_ methanation. Among these catalysts, the Ni-based catalytic system has been proven to be the most prominent because of the corresponding extraordinary activities and selectivity, as well as the relative lower costs (Gonçalves et al., 2022; Italiano et al., 2022; Ren et al., 2022; Xie et al., 2022). Nevertheless, the agglomeration of Ni metallic particles at a high temperature that inevitably results in catalyst deactivation has always been the bottleneck. Besides, at a low temperature, thermal sintering can also take place by reason of the interactions between Ni and CO, leading to the formation of nickel carbonyls followed by an increase in deactivation. Furthermore, there is a general consensus that the Ni-based catalysts are actually unfit for low-temperature hydrogenation reactions. However, for CO_2_ hydrogenation to methane, the process is in fact highly exothermic and profoundly affected by pressure and temperature, giving rise to contradictory requirements of reaction conditions for achieving both high CO_2_ conversion and methane selectivity (Boukha et al., 2022; Makdee et al., 2022; Pu et al., 2022). Consequently, in order to effectively keep from the thermodynamics and kinetics equilibrium limitations, a breakthrough in design and development of Ni-based catalysts with a conspicuous catalytic performance and robust stability at a low temperature is necessary and much awaited. Strategies with an eye on active metal, supports, and catalyst preparation methods have been frequently reported and have mainly included metal doping, encapsulation and alloying, metal–support interaction regulation, support design, modification, and morphology engineering, among which supports have carried considerable weightage in catalyst construction and fabrication, maximizing the corresponding catalytic performances and anti-sintering abilities without doubt (Yang et al., 2021). Oxide supports, for example, Al_2_O_3_, TiO_2_, SiO_2_, MgO, CeO_2_, ZrO_2_, and Nb_2_O_5,_ have been widely investigated and proved to be very active because of their characteristics, such as enhanced Ni dispersion, stable anchoring sites, suitable acid/basic properties, and an appropriate amount of structural defects (Zhou et al., 2016; Wang L. et al., 2020; Wang L. X. et al., 2020; Hu et al., 2021; Wang Z. M. et al., 2022; Zafar et al., 2022). Even though these advantages have indeed made contributions in regulating and controlling the compositions and structures of catalysts, the resulting catalytic activities and thermal stabilities can only be promoted to a limited extent. Thus, it is still highly desirable to keep digging into fairly promising Ni-based catalysts so as to boost the corresponding CO_2_ methanation activities and stabilities.

As one of the most popular supports for metal-based catalysts, zeolites with high specific surface areas, hydrothermal stabilities, regular channels, and typical ion-exchange and adsorption properties are of great academic and practical importance in catalysis (Wang X. Y. et al., 2022). For CO_2_ methanation reactions, certain kinds of zeolites have been reported, which include ZSM-5, USY, BEA, and MCM-41 adopted zeolites (Graca¸ et al., 2014; Gac et al., 2021; Hussain et al., 2021; Uttamaprakrom et al., 2021; Cui et al., 2022), in which a series of transition metals have been incorporated by means of co-crystallization, recrystallization, inter-zeolite transformation, encapsulation, and two-step post-synthesis preparation methods (Franken et al., 2020; Ra et al., 2020; Fan and Tahir, 2021; Frei et al., 2021; Hwang et al., 2021; Skrypnik et al., 2022). According to the encouraging results, the use of zeolite support for CO_2_ hydrogenation to methane did improve metal dispersion, metal–support interactions, and CO_2_ adsorption activations, resulting in promoted low-temperature kinetics and remarkable methane selectivity (Azzolina-Jury et al., 2017; Upasen et al., 2022). However, the evaluated zeolite catalysts were mostly treated with surface modifications, and their catalytic promotion have always been backed with other additives and metal promoters (Kosinov and Hensen, 2020; Liu et al., 2020; Zhang et al., 2020; Yang et al., 2021). Hence, there is plenty of scope for the development of zeolite-based catalysts with the inherent framework and physicochemical properties that are useful in and highly compatible with CO_2_ methanation processes.

ETS-10 zeolite, different from other widely used aluminosilicate zeolites such as MFI, FAU, and LTA, is a titanosilicate zeolite that is characterized by the unique three-dimensional 12-membered ring network and intrinsic Lewis basicity, which is derived from the specific corner sharing TiO_6_^2−^ octahedra with two negative charges (Xiang and Wu, 2019a; Chen et al., 2022). Besides, the interconnected channels of ETS-10 present a free entrance of about 0.8 × 0.5 nm, enabling the easy adsorption and diffusion of small molecules such as CO_2_, CO, CH_4_, H_2_, and H_2_O (Fu et al., 2018). As a result, due to the peculiar framework architectures and chemical compositions, as well as the relatively wide pore dimensions, ETS-10 has gained more and more attention and acquitted itself admirably in adsorption, ion exchange, and shape-selective catalysis. Actually, in previously published reports, the Ni-based ETS-10 catalyst has been employed for a hydrogenation process and proved to be conspicuously catalytically active with excellent selectivity and extraordinary stability in heterogeneous catalysis (Chen et al., 2016). Furthermore, considering the electron acceptor characteristics and geometric construction of the CO_2_ molecule, the typical structural unit of the -Ti-O-Ti- chain in ETS-10 that is characterized by extraordinary and strong electron donor capability deserves to place great expectations on boosting CO_2_ activation and conversion.

Hence, the main goal of this work was to construct catalysts made up of Ni active phased and structurally unique ETS-10 zeolite that is a self-contained Lewis base and can be highly envisioned to make an indispensable contribution to the selective conversion of CO_2_ into methane. Specifically, when compared to previously reported studies on the popular USY, ZSM-5, and beta zeolite-based catalysts, as well as those well-reviewed rare earth oxides catalysts, the prepared ETS-10 zeolite catalyst herein has been proven to be more potential in adsorption and activation of CO_2_. To account for this, intensive characterizations and measurements were carried out, which further helped to get deep insights into the corresponding catalysis promotion mechanisms. Furthermore, the impact of different Ni incorporation methods on catalyst stabilities has also been evaluated by relevant techniques.

## 2 Experimental

### 2.1 Catalyst preparation

A hydrothermal method was used with a molar composition of 1.0 TiO_2_: 7.1 SiO_2_: 4.4 Na_2_O: 1.9 K_2_O: 0.4 NiO: 160.0 H_2_O according to our previous studies (Xiang and Wu, 2019a). Typically, 6.0 ml of 6.67 mol/L aqueous NaOH was added into 10.0 ml aqueous water glass solution [SiO_2_/Na_2_O (molar ratio): 3.67] under vigorous stirring. Afterward, the mixed solution composed of 0.5 g nickel (II) nitrate hexahydrate and 7.0 g of TiCl_3_ solution (17 wt% in HCl) was introduced by slow dropwise addition. Then, 7.6 ml of 3.62 mol/L KF aqueous solution was added, followed by continuous stirring for 1.5 h. The obtained gel was subsequently transferred into a Teflon-coated stainless steel autoclave for crystallization (230°C, 64 h). The resulting product was then filtered, washed, and dried at 100°C overnight before being calcined in air at 450°C for 5 h. For comparison, incipient wetness impregnation was also performed with the ETS-10 zeolite prepared in the same way as abovementioned and by using parallel amounts of nitrate precursor. The resulting catalyst sample was named Ni/ETS-10. Other ETS-10-based catalysts with various metal species introduced by the doping method were also prepared and named M-ETS-10 (M refers to metal species).

Besides, a series of common zeolite supports was used to prepare Ni-based catalysts, which included Ni-ZSM-5, Ni-beta, Ni-SAPO-56, Ni-Y, and Ni-X. They were all prepared by the same method mentioned above, among which the ZSM-5, beta, Y, and X zeolite support were synthesized following previous works (Xiang et al., 2017; Vosoughi et al., 2021; Yu et al., 2022). For the Ni-SAPO-56 catalyst sample, its zeolite support was synthesized with a molar composition of 0.8 Al_2_O_3_: 0.9 SiO_2_: 1.0 P_2_O_5_: 40 H_2_O: 2.0 TMHD (template: N,N,N′,N′-tetramethyl-1,6-hexanediamine). The pseudoboehmite, phosphoric acid solution (85 wt%), and fumed silica were adopted as the aluminum, phosphorous, and silicon sources, respectively. Ni/SiO_2_ and Ni/Al_2_O_3_ were prepared by an incipient-impregnation method using parallel amounts of nitrate precursor.

### 2.2 Catalyst characterization

Powder X-ray diffraction (XRD) measurements were performed on a Rigaku powder X-ray diffractometer using Cu Kα radiation (λ = 0.1542 nm), and the scan range was from 5° to 45°. The actual Ni contents were determined by inductively coupled plasma optical emission spectrometry (ICP-OES) on a PerkinElmer 3300 DV emission spectrometer. N_2_ adsorption–desorption experiments were conducted on a Micromeritics ASAP 2020 M apparatus at −196°C. The samples were degassed at 300°C for at least 8 h prior to characterization. The specific surface area was calculated from the adsorption data using the Brunauer–Emmett–Teller (BET) equation. The pore size distribution was also obtained by using adsorption data and calculated according to the Barrett–Joyner–Halenda (BJH) model. The scanning electron microscopy (SEM) method was performed using an FEI Inspect F50. Transmission electron microscope (TEM) images were collected using a JEM-2100F.

The basicity of the catalysts was measured using temperature-programmed desorption of carbon dioxide (CO_2_-TPD) on a Micromeritics ASAP 2920 instrument, by which the corresponding CO_2_ adsorption capacity was also evaluated. A 200 mg sample was placed in a quartz tube and pretreated in a helium stream at 450°C for 2 h, and then cooled to 100°C to allow the CO_2_ gas to be passed over for 30 min. After the physically adsorbed CO_2_ was removed by flowing helium for 2 h at 100°C, the total flow rate of the gas was fixed at 10 m^3^/min, and the spectra were recorded from 100 to 650°C at a heating rate of 10°C/min. Similarly, H_2_-TPR measurements were also taken on this chemical adsorption instrument with the sample first being pretreated under Ar gas flow at 120°C for 1 h before being cooled down to room temperature. Then, a reducing gas of 10 vol% H_2_ in Ar reducing gas was introduced into the system, and the sample was heated to 600°C at a heating rate of 10°C/min. For the CO chemisorption experiment, the pretreatment process was carried out at 400°C in an H_2_ atmosphere for 2 h, after which He was purged and maintained for 1 h before the system was cooled to room temperature. Subsequently, a 10 vol% CO-He pulse was introduced and the corresponding CO uptake was measured by a TCD detector based on which the metal dispersion (*D*) was calculated using a previously reported equation (Xiang and Wu, 2019b).

*Quasi in situ* X-ray photoelectron spectroscopy (XPS) experiments were performed under vacuum on a Thermo Scientific ESCALAB 250Xi spectrometer with an Al Kα X-ray resource (hν = 1,486.6 ev). To avoid oxidization, a pretreatment with hydrogen at 400°C for all the samples in the reaction chamber was employed.

### 2.3 CO_2_ hydrogenation to methane

The catalytic performance evaluation of ETS-10–based catalysts for CO_2_ hydrogenation to methane was evaluated in a fixed bed reactor at atmospheric pressure. Before the reaction, the catalyst was reduced *in situ* in the H_2_ flow at 450°C for 3 h, after which the activated catalyst was exposed to the mixed reactant gas (CO_2_:H_2_:N_2_ = 1:4:1, molar ratio). The gas hourly space velocity (GHSV) was kept at 7,500 ml•g_cat_^−1^•h^−1^. The products were analyzed online by a GC-2060 gas chromatograph equipped with a flame ionization detector (FID) and a thermal conductivity detector (TCD). N_2_ was used as the internal standard for quantitative analysis. The CO_2_ conversion (X_CO2_) was calculated by using Eq 1:

$$X_{CO_{2}}=\frac{n_{CO_{2in}}-n_{CO_{2out}}}{n_{CO_{2in}}}\times 100\%,$$ (1)

where n_CO2in_ and n_CO2out_ are the moles of CO_2_ at the inlet and outlet, respectively. Also, the selectivity of CH_4_, CH_3_OH, and CO were calculated by the internal standard method based on Eqs 2–4:

$$S_{CH_{3}OH}=\frac{n_{CH_{3}OH_{out}}}{n_{CO_{2in}}-n_{CO_{2out}}}\times 100\%,$$ (2)

$$S_{CH_{4}}=\frac{n_{CH_{4out}}}{n_{CO_{2in}}-n_{CO_{2out}}}\times 100\%,$$ (3)

$$S_{CO}=\frac{n_{CO_{out}}}{n_{CO_{2in}}-n_{CO_{2out}}}\times 100\%,$$ (4)

where n_CH3OHout_, n_CH4out_, and n_COout_ refer to moles of CH_3_OH, CH_4_, and CO at the outlet, respectively.

The space–time yield (*STY*) of CH_4_ was expressed as grams of CH_4_ per hour and per gram of metal. The *STY*_CH4_ was calculated by the following equation:

$$STY_{CH4}=\frac{n_{CO_{2}in}\times X_{CO_{2}}\times S_{CH4}\times M_{CH4}}{m_{Ni}\times t},$$ (5)

where m_Ni_ is the mass of the Ni-based catalyst.

## 3 Results and discussion

### 3.1 Structure characteristics of ETS-10–based catalyst

**FIGURE 1:**
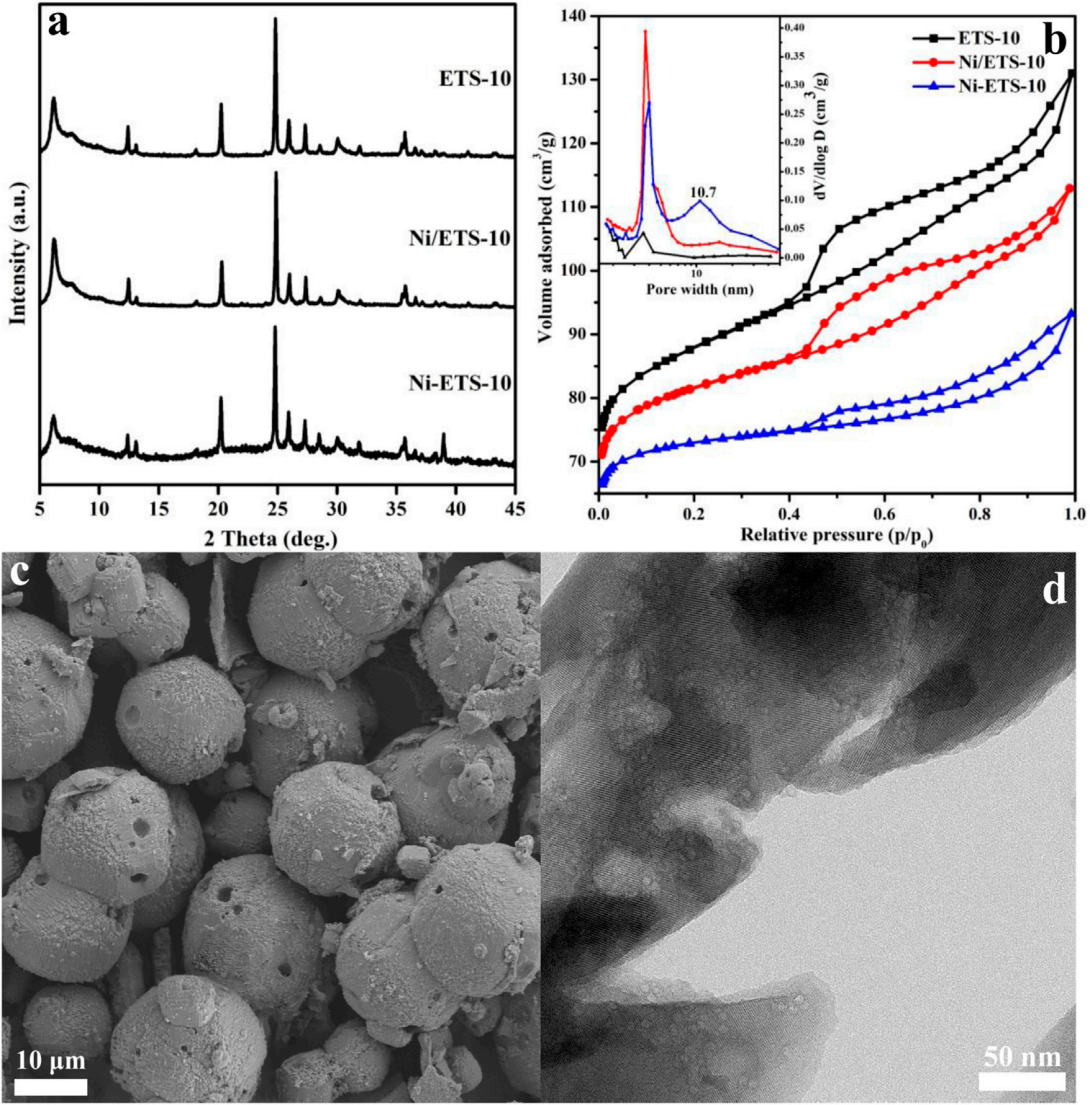
**(A)** XRD patterns and **(B)** N_2_ adsorption–desorption isotherm and pore size distribution (insert, calculated using desorption branch) of ETS-10–based samples; **(C)** and **(D)** SEM and TEM images of the Ni-ETS-10 sample.

As can be seen from Figure 1A, the XRD patterns of Ni-ETS-10 and Ni/ETS-10 display well-resolved peaks associated with the ETS structure in the range of 5°–45°, which is similar to that of the pristine ETS-10 sample but obviously with lower peak intensities. Indeed, by combining these results with N_2_ adsorption–desorption analysis data (Table 1), the decreased BET-specific area further confirms that the introduction of nickel species did threaten the framework integrity, leading to material crystallinity being reduced to different degrees. However, it is worth noting that the Ni particles were not presented in the XRD patterns for the two Ni-based ETS-10 catalyst samples, while according to the ICP analysis, the actual Ni contents were 3.7 wt% and 4.2 wt%, respectively, indicating the high dispersion of Ni species. Moreover, based on the CO-TPD results, the calculated dispersion of nickel species for Ni-ETS-10 and Ni/ETS-10 is 37.4% and 27.2%, which is no worse than that widely reported for Ni-based catalysts. Besides, when comparing Ni-ETS-10 with Ni/ETS-10, although both the calculated crystallinity and BET surface area were at a disadvantage, the introduced mesopores with a pore size distribution centered at 10 nm (Figure 1B) prove that they deserve the attention.

**TABLE 1:** Physicochemical properties of ETS-10–based samples.

Sample	Ni contents^a^	C^b^	*S*_BET_ ^c^	*S*_mic_ ^d^	*S*_ext_ ^e^	*V*_micro_ ^f^	*V*_meso_ ^g^	*D* ^h^	*d* ^i^
ETS-10	—	98.2	337	291	46	0.12	0.01	4	-
Ni/ETS-10	4.2	91.9	312	280	32	0.11	0.02	4	27.2
Ni-ETS-10	3.7	83.6	287	204	83	0.10	0.10	10	37.4

^a^ The actual Ni contents detected by ICP (wt%).

^b^ Crystallinity calculated by the Scherrer equation (%).

^c^ BET, surface area (m^2^/g).

^d^ Microporous surface area (m^2^/g).

^e^ External surface area, obtained from the *t*-plot method (m^2^/g).

^f^ Microporous pore volume, obtained from the *t*-plot method (cm^3^/g).

^g^ Mesoporous pore volume, obtained from BJH adsorption cumulative volume of pores between 1.7 and 300 nm in diameter (cm^3^/g).

^h^ Mean pore diameter (nm).

^i^ Dispersion of supported metal particles (%).

Figure 1C shows the SEM image of Ni-ETS-10 with smooth spherical particles of uniform size (17–21 μm). In addition, the obvious surface defect gives a visual illustration that the direct doping of Ni species influences the structural integrity of ETS-10 zeolite, leading to apparently declined crystallinity and BET-specific area, but with the hierarchical pores being formed. The high-magnification TEM image of the thin-sectioned Ni-ETS-10 is shown in Figure 1D. As can be seen, the numerous light areas strongly evidence the presence of abundant hierarchical pores, and the corresponding size range is also visibly uniform and in line with the pore size distribution resulting from N_2_ adsorption–desorption. On the other hand, the lattice fringes of Ni-ETS-10 crystals are basically intact, indicating that the native microporous structure delightfully survived the ravages of forming hierarchical pores.

**FIGURE 2:**
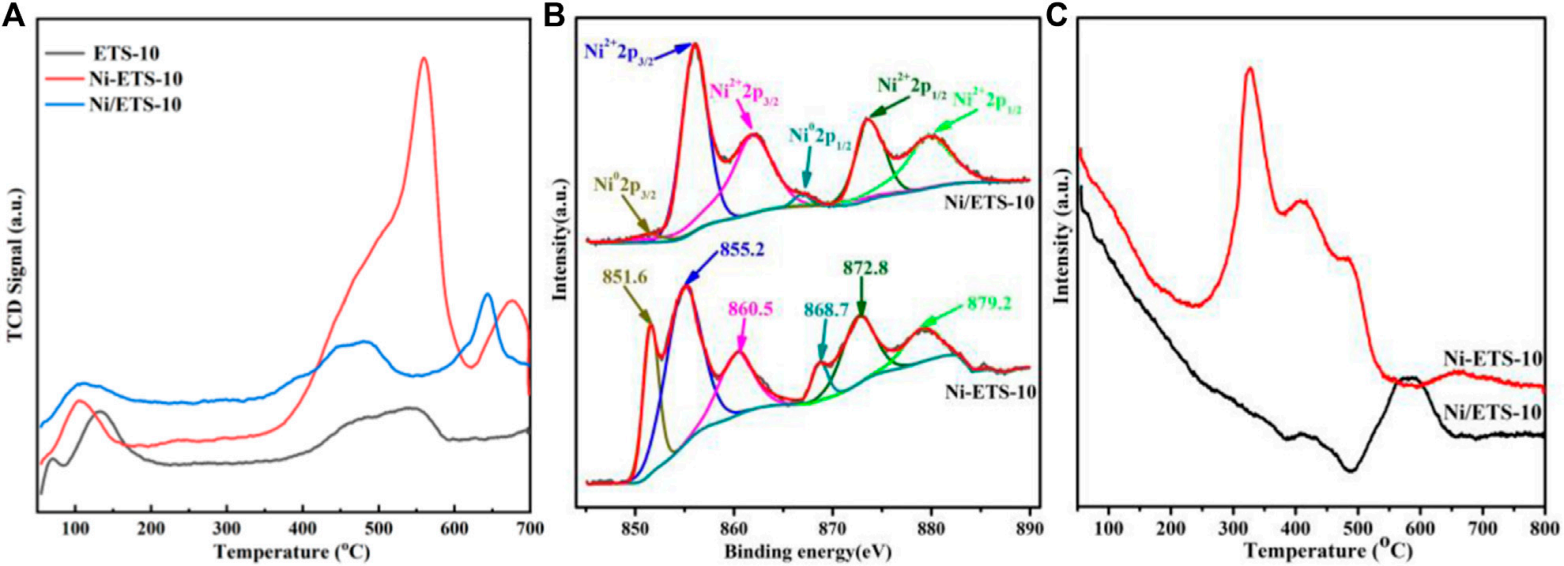
**(A)** CO_2_-TPD profiles of ETS-10, Ni-ETS-10, and Ni/ETS-10 samples; **(B)** XPS spectra of Ni 2p for Ni-ETS-10 and Ni/ETS-10 samples; **(C)** H_2_-TPR profiles of Ni-METS-10 and Ni/ETS-10 samples.

The basic properties of ETS-10, Ni-ETS-10, and Ni/ETS-10 were explored by CO_2_-TPD, and the results are shown in Figure 2A and Table 2. For all the three samples, even though the peak intensities were relatively weak, there is a non-negligible peak in the temperature range of 100°C and 150°C, which has resulted from the interactions between CO_2_ and the weak basic surface hydroxyl groups on ETS-10 zeolite-based samples. When comparing the results of ETS-10 and Ni/ETS-10, the significant difference was the dramatic shift of base strength distribution to high temperature rather than the modestly increased CO_2_ adsorption capacity, demonstrating that there are more high-strength basic sites in Ni/ETS-10 than that in the pristine ETS-10 sample. When it comes to the catalyst with nickel species introduced by *in situ* doping, not only did the total CO_2_ adsorption rise sharply to 1.29 mmol/g but it also presents mainly strong basic sites with the CO_2_ desorption being concentrated in the temperature range higher than 500°C. This corresponds well to our previous study that shows, other than the structure and pore topology, the presence of transition metal species and their composition, as well as the interaction with the zeolite support, all make significant contributions to enhance the basicity of ETS-10 zeolite–based catalysts (Xiang and Wu, 2018). Moreover, according to the comparison between Ni/ETS-10 and Ni-ETS-10, though with the same metal active phase, a definite edge in CO_2_ adsorption for the latter exists, especially in the high-temperature region, that further confirms the resultant hierarchical structure, the optimized metal dispersion (37.4% *vs.* 27.2%), and the interactions with zeolite supports which definitely endow Ni-ETS-10 with a great potential for CO_2_ methanation. In particular, the hierarchical structure existing in the zeolite channel system had long been considered as a promoter for exposing more catalytic active sites and offering easy access for metal species to zeolite supports. Thus, it is fair to say that the significantly improved basic strength of Ni-ETS-10 herein owed much to the introduced hierarchical structure.

**TABLE 2:** Basic properties of the ETS-10 zeolite samples.

Sample	Temperature of peak (^o^C)				CO_2_ adsorption (mmol/g)
ETS-10	68	129	478	540	0.76
Ni/ETS-10	109	469	644		0.86
Ni-ETS-10	106	506	562	677	1.29

The X-ray photoelectron spectroscopy (XPS) spectra were collected to investigate the surface composition and chemical bonding states of the active sites on the reduced Ni-based catalysts. As shown in Figure 2B, there are six characteristic peaks from the Ni 2p XPS spectra of both Ni-ETS-10 and Ni/ETS-10 catalysts. The peaks around 868.7 and 851.6 eV can be assigned to Ni^0^ 2p_1/2_ and 2p_3/2_ phases, respectively. The peaks appearing near 855.2 and 872.8 eV can be attributed to the presence of Ni^2+^ species at the zeolite exchange sites that strongly interacted with support. The other two multi-split peaks at ∼860.5 and 879.2 eV fell to the shake-up satellite peaks, which further indicate the location of Ni^2+^ species at the zeolite exchange sites. Besides, according to the Ni^0^/Ni^2+^ ratio of the summarized XPS results, Ni-ETS-10 unquestionably contained more Ni^0^ (26.1%) than Ni/ETS-10 (3.8%), indicating the presence of stronger metal–support interactions.

H_2_-TPR was used to further explore the behavior of surface Ni species and their interactions with the ETS-10 zeolite support. According to the H_2_-TPR results, there were three salient peaks around 327°C, 414°C, and 489°C for Ni-ETS-10, with a total H_2_ consumption of 0.69 mmol/g (Figure 2C). As reported previously, the reduction took place at relatively low temperatures, which can be assigned to those NiO species on the surface of ETS-10 being reduced to metallic Ni, illustrating their weak interactions with the zeolite support (Vosoughi et al., 2021). The peaks near 400°C were due to the moderately reduced NiO species deposited inside the porous structure of the ETS-10 zeolites. The peaks at higher reduction temperatures may be related to Ni^2+^ species that strongly interacted with the zeolite support. While for Ni/ETS-10, the reduction peak shifted to a higher temperature of 580°C with the onset temperature of 410°C with a total H_2_ consumption of 0.26 mmol/g, indicating its poorer reducibility and declined hydrogen dissociation ability. As a result, it can be concluded that doping can benefit the reducibility of nickel species, which is in line with the results obtained from the XPS spectra.

### 3.2 Catalytic activity

Three catalysts, ETS-10, Ni-ETS-10, and Ni/ETS-10, were first tested, and the results are listed in Table 3. Besides, a blank experiment with no presence of any catalyst was also conducted. The results showed that it failed to convert CO_2_ to methane under certain reaction conditions. When it came to those catalytic runs, it was obvious that the existence of catalysts did play a part in promoting CO_2_ activation and conversion. Furthermore, it seemed that except for the desired product methane, CO and methanol were the main interferential factors that went against the improvement of selectivity.

**TABLE 3:** Hydrogenation of CO_2_ on different catalysts^a^.

Catalyst	C^b^ (%)	S_CH4_ ^c^ (%)	S_CO_^d^(%)	S_methanol_^e^(%)
Blank	0	0	0	0
ETS-10	1.48	1.33	95.14	3.53
Ni/ETS-10	5.19	12.36	74.79	12.85
Ni-ETS-10	6.21	16.44	70.66	12.90

^a^ Reaction conditions: 0.2 g catalyst, 200°C, 3 h, CO_2_:H_2_:N_2_ = 1:4:1 (molar ratio), 3.0 MPa, and GHSV = 7,500 ml•g_cat_^−1^•h^−1^.

^b^ Conversion of CO_2_.

^c,d,e^ Selectivity of CH_4_, CO and methanol, respectively.

Compared with the pristine ETS-10 catalyst sample, the introduction of Ni species was definitely beneficial to both CO_2_ conversion and product selectivity. Meanwhile, according to the comparison between Ni-ETS-10 and Ni/ETS-10, the superiority of Ni-ETS-10 in facilitating CO_2_ transformation and methane production was noticeable, which can be attributed to the enhanced molecule transfer, exposure of active sites, and metal dispersion that resulted from the hierarchical structures. Thus, Ni-ETS-10 was chosen for further investigating the processes of CO_2_ hydrogenation to methane in the following experiments.

**FIGURE 3:**
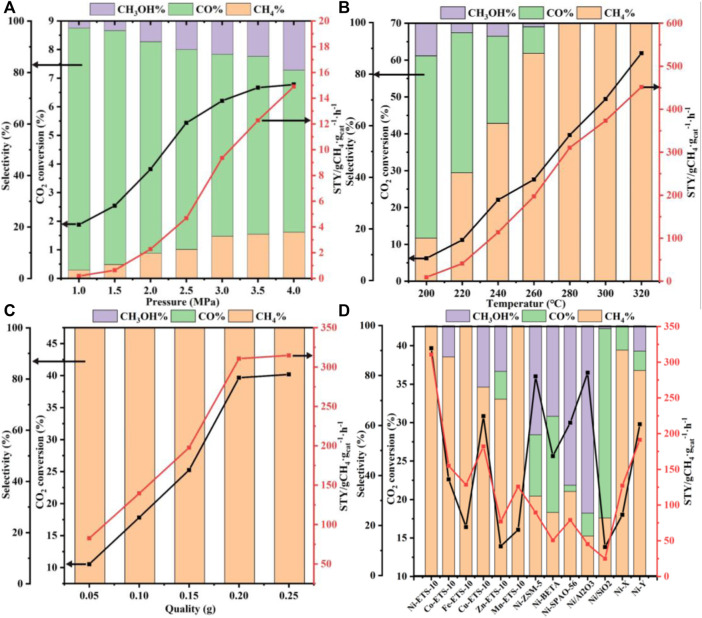
Activity of CO_2_ hydrogenation into methane over Ni-ETS-10 as a function of **(A)** pressure, **(B)** temperature, **(C)** catalyst quality, and **(D)** the activity of different catalysts for CO_2_ methanation (reaction conditions: 280°C, 3 MPa, 7,500 ml•g_cat_^−1^•h^−1^, 3 h, and 0.2 g catalyst).

Figure 3 shows the CO_2_ conversion, product selectivity, and *STY* value as a function of reaction pressure, temperature, and catalyst dosage and types. Considering that hydrogenation of CO_2_ to methane is in fact a molecule-reducing reaction, increasing the pressure is theoretically in favor of CO_2_ conversion. Indeed, based on the experimental data shown in Figure 3A, not only CO_2_ conversion but also methane selectivity increased with the increase of pressure. When the pressure was increased to 3.0 Mpa, the conversion of CO_2_ went up to 6.21%, and the selectivity of methane rose to 16.44%, after which a deceleration of growth took place. Consequently, as the reaction pressure continued to be increased to 3.5°Mpa, only 0.46% more CO_2_ conversion and 0.79% more methane selectivity were obtained. That is to say, when the pressure is over 3.0°MPa, further optimization is conducive but makes little sense in practice, especially taking the security and cost requirements into account.

As a typical exothermic reaction, the hydrogenation of CO_2_ to methane deservedly benefits from elevated temperature, which can contribute to the activation of those molecules with low energy. Thus, effective collision and bonding among reactant molecules can be greatly enhanced, giving rise to an accelerated reaction rate. It can be seen from Figure 3B that the transformation of CO_2_ to methane could be markedly boosted with a higher temperature. And notably, when it rose to 280°C, CO_2_ was transformed to methane completely, leading to the desired selectivity of 100%. More importantly, the selectivity is maintained at 100% when the reaction temperature is raised to 300°C and then to 320°C. Meanwhile, the continuous growth of both CO_2_ conversion and space–time yield of CH_4_ are observed in the temperature range of 200°C–320°C. However, considering the actual energy consumption required for a high operating temperature, a comparatively low temperature (<300°C) seems to be more suitable. Thus, the following experiments were carried out under 280°C.

It is well known that the amount of catalyst definitely plays a part in improving the catalytic performance by adjusting its active sites. Typically, the more the catalysts are added, the more the active sites become available for reactants' adsorption. As shown in Figure 3C, increasing the catalyst dosage from 0.05 to 0.20 g can bring about a growth leap in CO_2_ conversion and *STY*_*CH4*_, after which the rising tendency begins to level off. This can be demonstrated as access to more and more indispensable active sites that significantly promotes the activation of CO_2_ and facilitates subsequent combinations between the activated-CO_2_ intermediates and the adsorbed-dissociated H_2_, and then accelerates the formation of methane. Despite being conducive to enhancing catalytic activities, there appears no need to blindly increase the quantity of catalysts once it has reached up to 0.2 g, where the catalytic active sites in the reaction system are saturated. What is noteworthy is that no matter how the catalyst quality is regulated, the resulting methane selectivity remains stable at 100% under the current reaction conditions.

**SCHEME 1:**
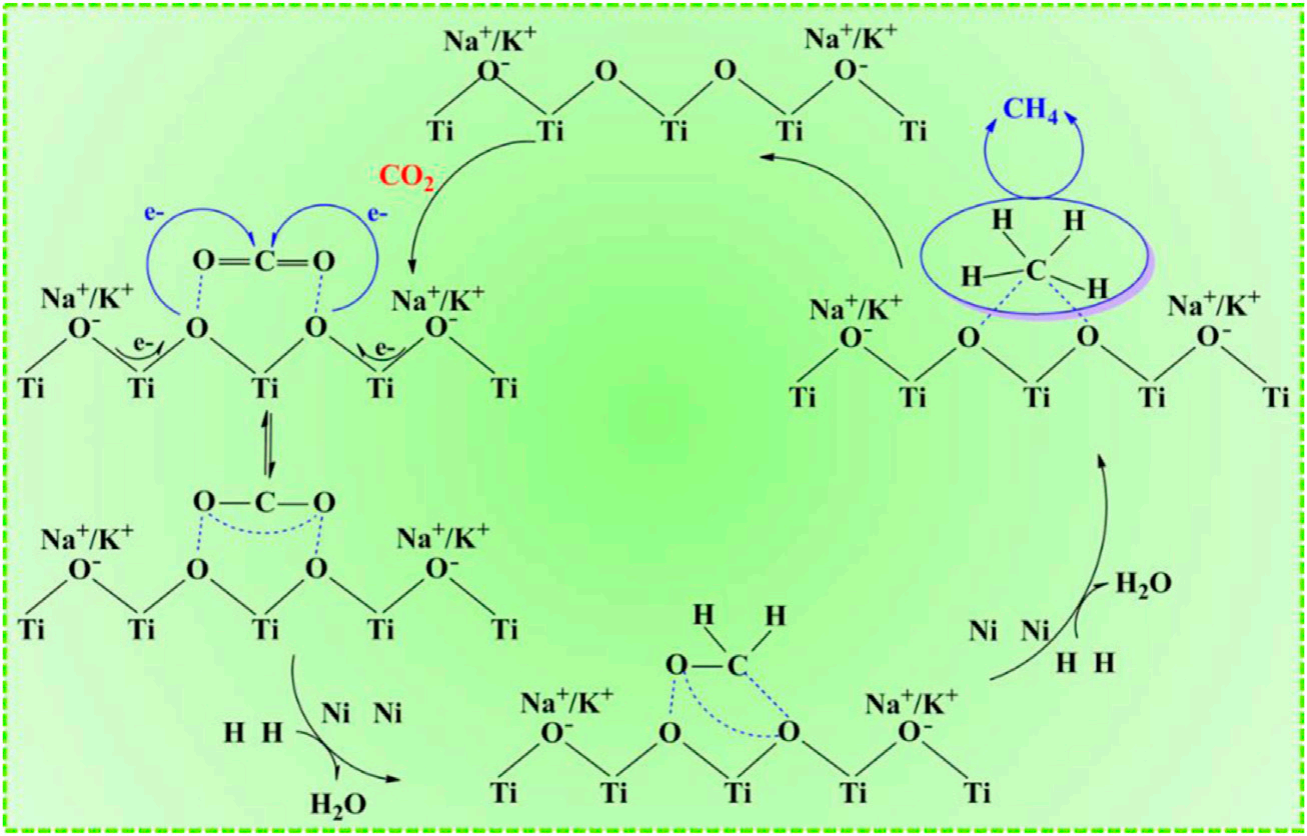
Possible reaction mechanism for CO_2_ hydrogenation into methane over Ni-ETS-10.

The impact of the catalyst types involving different supports and active metal species on CO_2_ conversion and methane selectivity was also taken into consideration and explored under the reaction conditions of 280°C, 3 MPa, 7,500 ml•g_cat_^−1^•h^−1^, 3 h, and 0.2 g catalyst. According to the comparison between the Ni-ETS-10 and other catalysts (Figure 3D), the superiority in CO_2_ conversion (39.7%) and methane selectivity (100%) was distinct. For Cu-ETS-10, though it gave good results in CO_2_ activation and transformation, the moderate H_2_ adsorption–dissociation capacity of copper species resulted in a relatively strong appetite for methanol formation. On the contrary, both Fe-ETS-10 and Mn-ETS-10 catalysts were highly enthusiastic about producing methane but failed to effectively promote the transformation of CO_2_. For the remaining two ETS-10 zeolite–based catalysts, Co-ETS-10 and Zn-ETS-10, their catalytic performances were too common to be on par with others. Apart from the reactivity discrepancies caused by various active metals, the differences caused by diverse catalyst supports, such as ZSM-5, beta, SAPO-56, X, Y, Al_2_O_3_, and SiO_2_ that have been widely reported and used for CO_2_ conversion were investigated. Ni-ZSM-5 and Ni/Al_2_O_3_ were undoubtedly two of the best for CO_2_ activation, which shows comparative CO_2_ conversion with Ni-ETS-10. However, there was still an obvious imperfection of product selectivity, which appeared in the intensive formation of CO and methanol. Ni-X was better in obtaining target product methane with a higher selectivity of 90.2%, but unfortunately, it could not activate CO_2_ effectively. Also for the Ni-Y catalyst, the corresponding catalytic performance was a middle case with the CO_2_ conversion approaching 30% (29.8%) and methane selectivity just above 80% (82.0%). Even though Ni-beta and Ni-SAPO-56 did not seem good for the catalytic conversion of CO_2_ to methane, they were still superior to Ni/SiO_2_, over which only a small amount of CO_2_ (13.8%) was converted and the main product was CO. So, there are reasons to believe that the employment of a suitable catalyst support is of great importance for CO_2_ activation and oriented conversion. The three most common aluminosilicate zeolite catalysts, ZSM-5, beta, and Y, which are characterized by strong surface Brønsted acidity/basicity, have been widely used in various catalytic reactions, especially hydrogenation reactions. However, precisely because of this typical feature, product selectivity is always the key bottleneck for further applications. As for the two representative commercial catalysts, Ni/Al_2_O_3_ clearly outperforms Ni/SiO_2_ due to the presence of abundant surface Lewis acid that endows it with excellent capability for CO_2_ adsorption and activation. Being different from those conventional silicon aluminum zeolites, X zeolite with inherent basicity is more prone to methane formation. However, the base strength is far from enough for high efficiency. Thus, it follows that the ability and method of CO_2_ adsorption–activation are the determinants for the succeeding hydrogenation process and producing methane, which are closely linked to the physicochemical properties and structure of the catalyst supports. Besides, the active metal species and their composition, dispersion, and corresponding interaction with the support all dominated the catalytic performance. Herein, Ni-ETS-10 is, of course, the most outstanding catalyst for the hydrogenation of CO_2_ to methane. This can be due to the unique 3D pore structure and framework of ETS-10 zeolite, especially the peculiar structure unit of the -Ti-O-Ti- chain that is characterized by extraordinary and strong donor capability. Furthermore, the constructed multiple catalytic active centers those resulted from the desired interactions between the Ni species and ETS-10 framework do meet the demands of CO_2_ activation, which in fact can be promoted by making full use of its electron acceptor characteristic and geometric construction (Scheme 1).

### 3.3 Catalyst stability

**FIGURE 4:**
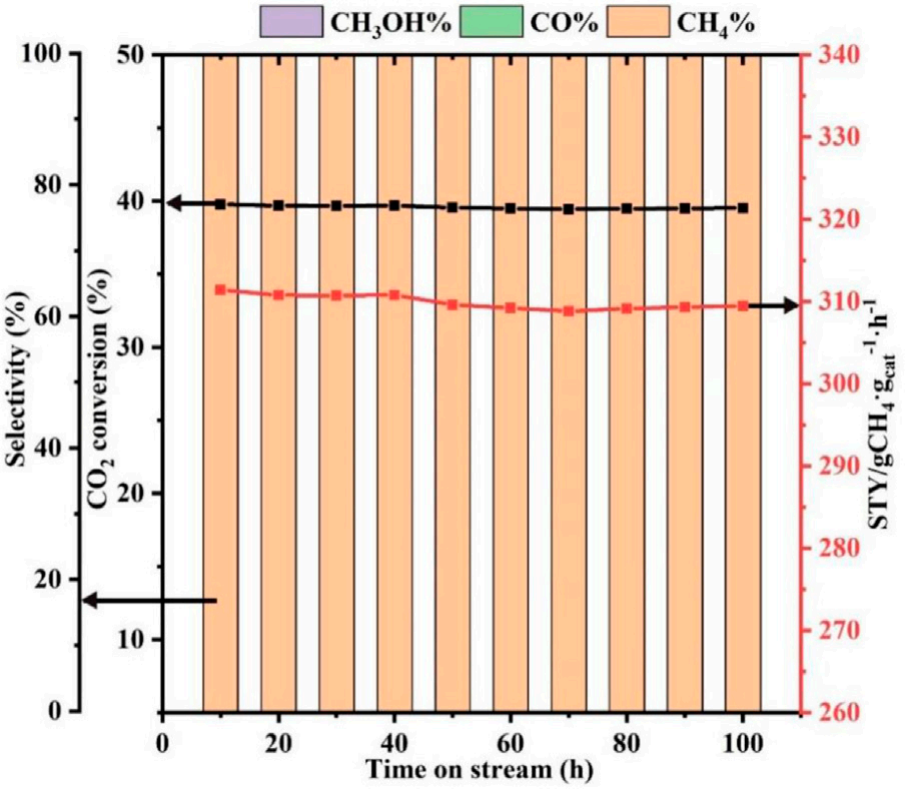
Stability of Ni-ETS-10 catalyst (reaction conditions: 280°C, 3 MPa, 7,500 ml•g_cat_^−1^•h^−1^, 3 h, and 0.2 g catalyst).

Catalyst stability is an important basis for practical applications. Hence, the stability of the Ni-ETS-10 catalyst after 100 h of reaction under the desired conditions was evaluated. As revealed in Figure 4, Ni-ETS-10 is proven to be reasonably stable at 280°C for 100 h with less than 5% decline in CO_2_ conversion and *STY*. More importantly, the methane selectivity can be sustained at a consistent level of 100% throughout the process under the optimized reaction conditions.

## 4 Conclusion

In conclusion, ETS-10 zeolite–based catalysts were used to investigate the process of methane formation from CO_2_ hydrogenation in detail. For comparison, different metal species and supports were studied, among which the Ni-ETS-10 catalyst prepared by the *in situ* doping method presented obvious advantages in both CO_2_ conversion and methane selectivity. This can be due to the exposure of more basic sites and the absence of porous blockage and structural damage by metal incorporation to a great extent, which is because of the introduced hierarchical pores. More importantly, the presence of more highly dispersed Ni^0^ species is vital for accelerating the access of reactants to the Lewis basic sites (TiO_6_^2−^). As a result, the reduced Ni-ETS-10 with better metal dispersion, advantageous hierarchical structure, and stronger Lewis basic strength has been proven to be highly reactive among the studied catalysts for CO_2_ hydrogenation, giving a 39.7% CO_2_ conversion and 100% methane selectivity under mild conditions. Last but not least, the catalytic performance of Ni-ETS-10 that was maintained at 280°C for 100 h with only less than 5% decline in CO_2_ conversion and *STY* indicated its excellent stability and reusability.

## Data Availability

The original contributions presented in the study are included in the article/Supplementary Material; further inquiries can be directed to the corresponding authors.
